# Acute and subchronic *in-vivo* effects of *Ferula hermonis* L. and *Sambucus nigra* L. and their potential active isolates in a diabetic mouse model of neuropathic pain

**DOI:** 10.1186/s12906-015-0780-7

**Published:** 2015-07-30

**Authors:** K. Raafat, A. El-Lakany

**Affiliations:** Department of Pharmaceutical Sciences, Faculty of Pharmacy, Beirut Arab University (BAU), 115020 Beirut, Lebanon

**Keywords:** *Ferula hermonis* L, *Sambucus nigra* L, *in vivo* antioxidant, *Diabetes mellitus*, Tactile allodynia, Thermal hyperalgesia

## Abstract

**Background:**

The prevalence of *Diabetes mellitus* (DM) is escalating rapidly worldwide, and associated with micro- and macrovascular complications. Diabetic neuropathy (DN) is a common complication of DM, and has a few approved therapies with limited efficacy and several side-effects. Herbal medicine is used worldwide as an effective alternative-medicine. The present study aims to investigate the activities of *Ferula hermonis* Boiss. EtAc (Ferula) and *Sambucus nigra* L. aqueous (Elder) extracts, and their potential active isolates; for acute (6 h) and subchronic (8 days) glucose homeostasis, *in vivo* antioxidant potential and DN amelioration in alloxan-induced DM mice model.

**Methods:**

DM was induced experimentally by injection of freshly prepared alloxan every 48-h for three times at a dose of 180 mg/kg. Utilizing tail-flick, hot-plate latencies (accessing thermal hyperalgesia) and von Frey filaments test (accessing tactile allodynia), DN was evaluated for longer period of time (8 weeks).

**Results:**

The most active isolates from Ferula was ferutinin, and Kaempferol from Elder utilizing bio-guided fractionation and RP-HPLC steeping methods. Compared to glibenclamide (GB) and tramadol (TRA), as positive controls, the highest doses of tested compounds exerted remarkable hypoglycemic and antinociceptive activities. The best acute hypoglycemic effect was observed with ferutinin (1.4 folds more effective than GB). Elder has shown the best subchronic hypoglycemic effect (2.6 folds more effective than GB) and the greatest efficacy against tactile allodynia following a single-administration, yet required repeated administration for improvement of thermal hyperalgesia.

**Conclusions:**

Without the use-limiting-side-effects of existing therapies, Ferula, Elder and their active isolates have shown significant results in ameliorating DM and long standing diabetes-induced complications.

## Background

Internationally, millions of people in the developing countries depend on medicinal plants as a primary source of healthcare [1]. Around 70,000 plants species are known to be used in folk and modern medicinal systems all over the world [2]. The international market of herbal plants is estimated to be US $ 62 billion which is suspected to grow to US $ 5 trillion by the year 2050 [1].

Owing to their biomedical properties, medicinal plants are extensively used in the management and prevention of age-related diseases, cardiovascular ailments, DM and related complications [3].

*Ferula* genus comprises more than 70 species, among them *Ferula hermonis* (Ferula) or Zallouh roots. Ferula has been recognized for its aphrodisiac powers for many years, and it grows in the Hermon Mountain of Lebanon. Several Ferula are appreciated in the Lebanese traditional medicine for the treatment of skin infections, stomach disorders, fever, dysentery, aphrodisiac and neurological disorders like hysterias [4]. More recently, cytotoxic and cancer preventing properties have been also investigated in plants and related to sesquiterpene derivatives, mainly to those of the daucane type, such as ferutinin (Ft) [5, 6]. Ferula is currently attracting an increasing interest in view of its content, and its additional biological benefits, besides its aphrodisiac interest. It would be expected that Ferula has effect on DM management as it has been used before in weight reduction and diabetes therapy mixture [7, 8]. Nevertheless, other *Ferula* species were briefly screened before solely for their hypoglycemic activity [8, 9]. Till date, Ferula has not been thoroughly investigated for its acute and subchronic effects on *Diabetes mellitus* (DM) and diabetic neuropathy (DN).

The inflorescences of *Sambucus nigra* L. (Elder) has long been known in herbal medicine in Asia, North Africa, Europe and America. Phytopharmacological data shows that Elder is rich in phenolic compounds, comprising phenolic acids, flavonoids, catechins, and proanthocyanidins [10]. Nevertheless, they exhibit anticancer, immune-stimulating [11], antibacterial, antiallergic, antitussive, bronchodilatory, and antiviral activity [12]. It would be expected that Elder has effect on DM management as it has been recognized alone or in combination for management of DM [13, 14]. Moreover, Elder polyphenols has been briefly assessed for its effect as a new approach on management of chronic metabolic diseases [15]. Till date, Elder has not been thoroughly investigated for longer time therapy on amelioration of DM and DN.

Oxidative stress is significantly related to the etiology of several diseases such as DM, cancer and cardiovascular disorders [16]. Consequently, it is important to enhance the body antioxidant potential for fighting against oxidative stress. Therefore, it has been broadly advised that individuals increase their intake of herbal plants rich in natural antioxidants in order to reduce the risk of various diseases [17, 18].

Nutritional habits and sedentary living standards of the modern societies leads to exponential rise in the prevalence of DM, which is estimated to reach the rate of 300 million cases by 2030 [19]. Present estimates indicate a 69 % increase in the number of adults in the developing countries that would be acquire DM between (2010–2030), compared to 20 % for developed countries [20]. An increasing risk of developing DM, which is a consequence of a combination of pathological conditions known as metabolic syndrome, which includes obesity, hyperglycemia, glucose intolerance and dyslipidemia [21]. DM is accompanied with diverse complications such as retinopathy, neuropathy, nephropathy, cardiomyopathy, vasculopathy, dermatopathy and encephalopathy [22]. DN has been defined as the occurrence of symptoms of peripheral nerve dysfunction in diabetics [23]. DN is characterized by pain, allodynia, hyperalgesia, paraesthesia, and affects ca. 50 % of people with significant morbidity, mortality and inferior quality of life [24].

Literature review indicated that no detailed study has been carried out to determine the efficacy of Ferula and Elder in the modulation of oxidative stress associated with DM and DN in experimental animals. Accordingly, the aim of the present work involves the study of possible hypoglycemic, *in vivo* antioxidant and diabetic neuropathy management effects of Ferula and Elder extracts and their potential active isolates.

## Methods

### Plant material

*Ferula hermonis* L. (Ferula) roots and *Sambucus nigra* L. (Elder) inflorescence, voucher specimens (PS-14-16) and (PS-14-21) respectively, were purchased commercially (Ibn-Al-Nafess, Lebanon) and dried specimens were deposited in the Faculty herbarium. The plant materials were authenticated by Prof. J. Habib (LU, Lebanon) with reference samples.

### Preparation of plant extracts

Ferula was extracted using ethyl acetate (EtAc). While, Elder was macerated in distilled water at room-temperature. The residues were removed by filtration. Both extracts were dried separately in a rotary-evaporator (Buchi, Germany) at temperature 40 °C under-vacuum [25, 26].

### Animals

All animal care and experiments were done on male Swiss-Webster-mice as described before [25, 27, 28]. Briefly, male Swiss-Webster mice (BAU, Lebanon) were accommodated for one week before the experimentation and caged with a 12-h light/dark cycle. All experiments were done abiding by principles of NIH-laboratory animal care [29], as well as the animal experiment legislation and with approval of the BAU Institutional Review Board (2014A-008-P-R-0010).

### Experimental procedure

#### Diabetes induction

DM was induced by a method defined before [25, 26]. Briefly, i.p. injection in mice of freshly prepared alloxan (Sigma-Aldrich, Germany) in mice (20–28 g) dissolved in saline every 48-h for three times at a dose of 180 mg/kg. Fasting glucose levels in the blood samples acquired from the tail of each mouse 72-h after the last alloxan injection were measured with Accu-chek Active™ Test Meter (Roche, USA). The mice with blood glucose level higher than 200 mg/dL were regarded to be diabetic and were used in the experiments [30].

#### Acute effect of extracts and their potential active isolates in alloxan-induced diabetic mice

The diabetic mice were divided into fourteen groups (7-animals/group). Group I received only saline, i.p. and served as control. Group II received glibenclamide (GB) as reference drug (5 mg/kg, i.p.) dissolved in DMSO. All other test compounds were dissolved in saline (vehicle). The Ferula extract was administered at the doses of 12.5, 25 and 50 mg/kg i.p. to the animals of group III, IV and V, respectively. Ft was administered at the doses of 0.4, 0.8 and 1.6 mg/kg i.p. to the animals of group VI, VII and VIII, respectively . The Elder extract was administered at the doses of 50, 100 and 200 mg/kg i.p. to the animals of group IX, X and XI, respectively. Kaempferol (KMF) was administered at the doses of 16, 32 and 64 mg/kg i.p. to the animals of group XII, XIII and XIV, respectively . Blood samples were collected from the tail just prior to and at 0.5, 2 and 6 h after dosing and blood glucose levels were determined.

#### Subchronic effect of extracts and their potential active isolates in alloxan-induced diabetic mice

The antidiabetic action were also tested during a longer duration. The mice were divided into groups containing healthy and diabetic animals. Group I (healthy mice, *n* = 7) received only vehicle i.p. for 7 days and served as control [30]. The diabetic mice were divided into fourteen groups (II–XV) of seven animals each. Group II received only saline i.p. for 7 days and served as diabetic control. Group III received glibenclamide as reference drug (5 mg/kg, i.p.) dissolved in vehicle for 7 days (positive control). The Ferula extract was administered at the doses of 12.5, 25 and 50 mg/kg i.p. to the animals of group IV, V and VI, respectively. Ft was administered at the doses of 0.4, 0.8 and 1.6 mg/kg i.p. to the animals of group VII, VIII and IX, respectively. The Elder extract was administered at the doses of 50, 100 and 200 mg/kg i.p. to the animals of group X, XI and XII, respectively. KMF was administered at the doses of 16, 32 and 64 mg/kg i.p. to the animals of group XIII, XIV and XV, respectively. Blood samples were gathered from the tail at 1st, 3rd, 5th, and 8th days after each treatment. Blood glucose, catalase levels, and animals body-weights were measured.

### Management of diabetic neuropathy

After 6-weeks of DM-induction, DN success rate (i.e., loss of sensory of thermal sensitivity significantly below 10S [31]) was ca. 80 %, and their neurological function were tested at one week intervals for 8 weeks, with tramadol (TRA) 10 mg/kg as a positive control; using:

#### Hot plate test

Hot plate analgesia meter (Ugo Basile, Italy) was employed for assessment of management of DN. The animals were put one at a time on a hot plate that is maintained at a temperature of 55 ± 0.1 °C. Response latency to jump or a hindpaw-lick was monitored utilizing an electronic-timer [25, 32].

#### Tail flick test

Tail-flick apparatus (Hugo-Sachs-Elektronik, Germany) was also used to evaluate the management of DN, using a method described before [25]. Briefly, a beam of light was focused on the dorsal surface of the mouse tail and the time until the tail flicked was monitored (tail-withdrawal latency) [32].

#### Von Frey filaments test

Tactile allodynia was assessed in mice by measuring paw withdrawal thresholds using calibrated von Frey filaments (*OptiHair*™, *Marstock* Nervtest™, Germany) utilizing a method described before [33]. In brief, filaments with increasing stiffness (0.5–45.3 g) were applied perpendicularly to the plantar surface of the mice-paw using an up–down method. A force just sufficient to bend the filament was applied for 5 s, and a positive response was assumed when abrupt withdrawal were exhibited.

### *In vivo* antioxidants estimation

Serum catalase (CAT) activity was determined utilizing the modified method described before in literature [34]. CAT activity was measured as kU/l.

### Statistical analysis

All values are presented as means ± S.E.M. Statistical differences between the treatments and the controls were tested by one-way analysis of variance (ANOVA) followed by the Student–Newman–Keuls test, for all tests except von Frey filaments was followed by Tukey’s HSD test, using the “OriginPro” statistic computer program [35]. A difference in the mean values of *p* < 0.05 or less was considered statistically significant.

### Bio-guided chromatographic fractionation and identification of the effective compound(s)

Ferula and Elder extracts were fractionated separately, using RP-column chromatography. Elution was done using ethanol: water (50:50) for Ferula extract, and EtAc, formic acid, water and hexane at ratios of (70:7.5:7.5:15) respectively as a mobile phase for Elder extract and eluent was collected in a series of fractions by time and examined the same way as the test solutions in this study. The active fraction was analyzed using RP-HPLC. HPLC analysis was carried out in a JASCO-instrument (JASCO, Japan). Methanol/ phosphate buffer 34.1 mM, pH 2.1, (50:50) for Ferula and Elder extracts and 1 ml/min flow rate. An RP-C18 endcapped Lichrospher column (250 × 4.6-mm I.D.; 5-μM particle-size) was employed (Merck, Germany), at 40 °C and UV-detector were adjusted to 254 nm and 370 nm for Ferula and Elder extracts, respectively. Serial dilutions of standards (5–100 ppm) solutions were utilized for preparing calibration curves.

## Results

### Bio-guided chromatographic fractionation and identification of the effective compound(s)

**Fig. 1:**
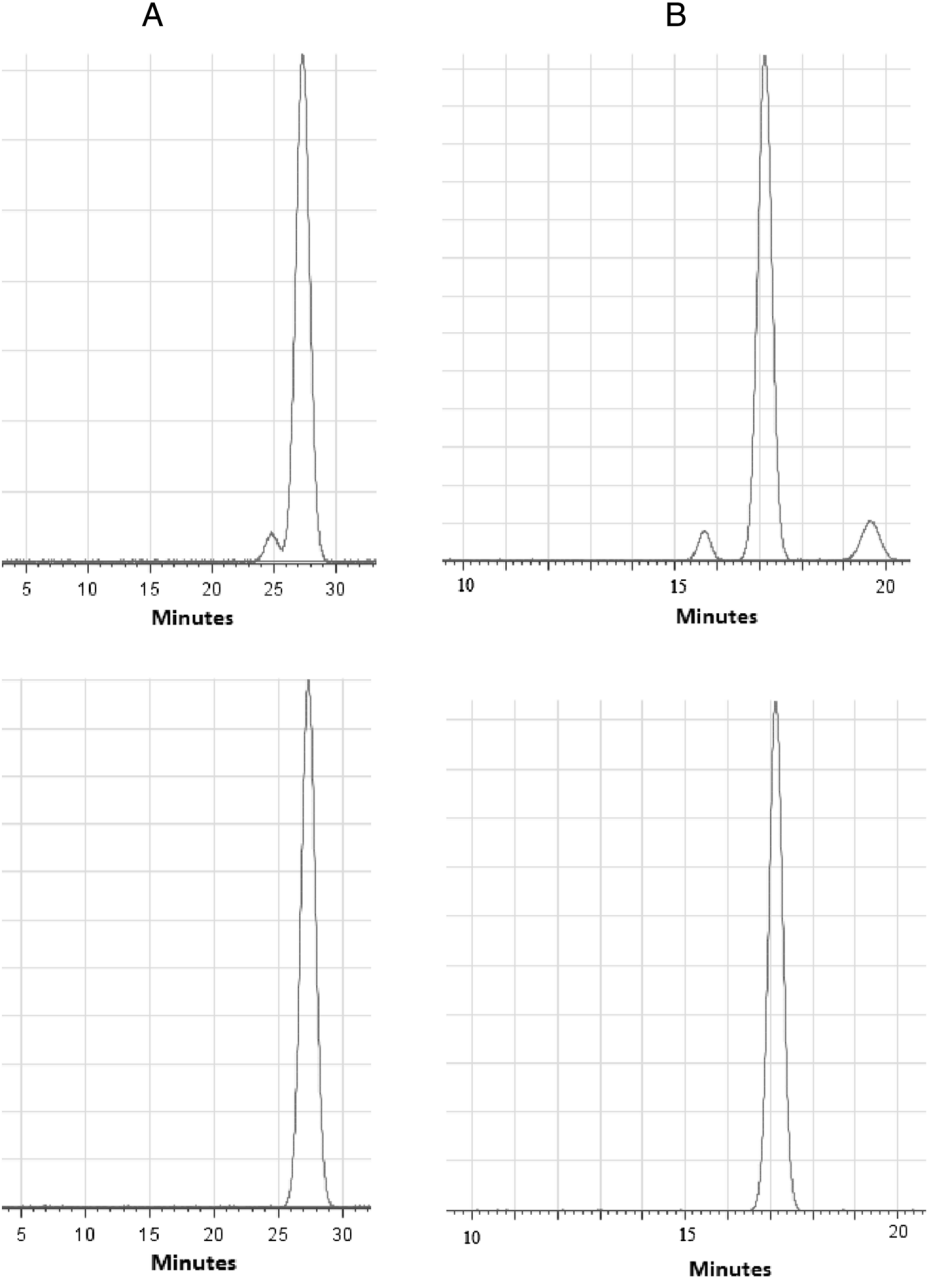
HPLC chromatogram utilizing C-18 reversed phase column and mobile phase was MeOH: phosphate buffer pH 2.41 (50:50) and flow rate 1.0 ml/min **a** the upper panel: Isolated ft, the lower panel: Standard ft. Measured at 254 nm. **b** the upper panel: Isolated KMF, the lower panel: Standard KMF. Measured at 370 nm

After the bio-guided fractionation, the most active Ferula extract fraction was injected into the HPLC instrument to study its pattern. The most active fraction major peak was Ferutinin (Ft) (6.6 %). Isolated Ft was identified using Ft standard steeping method (Fig. 1a). On the other hand, the most active Elder extract fraction major peaks were; Quercetin (45.6 %), Kaempferol (KMF) (32.0 %) and isorhamnetin (22.4 %). Isolated KMF was identified using KMF standard steeping method (Fig. 1b). Nevertheless, KMF was the most active isolated compound from Elder extract utilizing *in vivo* alloxan-diabetic mice.

**Fig. 2:**
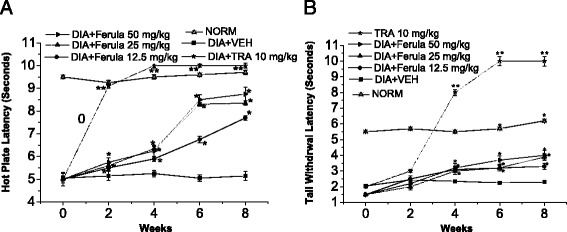
Effect of *F. hermonis* EtAc extract (Ferula) and Tramadol (TRA) 10 mg/kg, as positive control, on the hot plate and tail withdrawal latencies in alloxan-induced diabetic mice. **a** Hot plate latency: (*Crossed-triangles, straight line*) NORM: normal control mice. (*Closed-squares, straight-line*) DIA + VEH: diabetic animals treated with vehicle as control. (*Solid-stars, dotted-line*) Positive control TRA 10 mg/kg: alloxan treated mice with TRA 10 mg/kg. (*Solid-circles, straight-line*) DIA + Ferula 12.5 mg/kg: diabetic animals treated with Ferula 12.5 mg/kg. (*Up-triangles, dashed-line*) DIA + Ferula 25 mg/kg: diabetic animals treated with Ferula 25 mg/kg. (*Right-triangles, dashed-dotted-line*) DIA + Ferula 50 mg/kg: diabetic animals treated with Ferula 50 mg/kg. **b** Tail withdrawal latency: (*Crossed-triangles, straight line*) NORM: normal control mice. (*Closed-squares, straight-line*) DIA + VEH: diabetic animals treated with vehicle as control. (*Solid-stars, dotted-line*) Positive control TRA 10 mg/kg: alloxan treated mice with TRA 10 mg/kg. (*Solid-circles, straight-line*) DIA + Ferula 12.5 mg/kg: diabetic animals treated with Ferula 12.5 mg/kg. (*Up-triangles, dashed-line*) DIA + Ferula 25 mg/kg: diabetic animals treated with Ferula 25 mg/kg. (*Right-triangles, dashed-dotted-line*) DIA + Ferula 50 mg/kg: diabetic animals treated with Ferula 50 mg/kg. Data are expressed in mean ± S.E.M. “*” means *P* < 0 .05 compared with vehicle.“**” means *P* < 0 .01 compared with vehicle

**Fig. 3:**
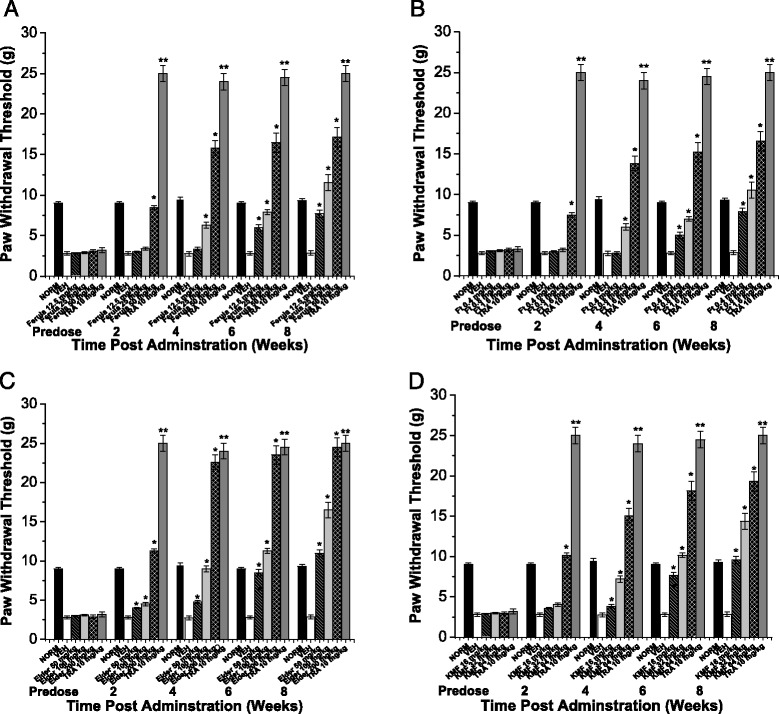
Effect of *F.hermonis* EtAc extract (Ferula), Ferutinin (Ft), *S.nigra* aqueous extract (Elder), Kaempferol (KMF) and Tramadol (TRA) 10 mg/kg, as a positive control, on tactile allodynia in neuropathic model in alloxan-induced diabetic mice **a** Ferula group: paw withdrawal thresholds to von Frey filaments were determined on hind paw prior to (Predose) and up to 8 weeks following i.p. injection of (12.5, 25 and 50 mg/Kg) Ferula **b** Ft group: paw withdrawal thresholds to von Frey filaments were determined on hind paw prior to (Predose) and up to 8 weeks following i.p. injection of (0.4, 0.8 and 1.6 mg/Kg) Ft. **c** Elder group: paw withdrawal thresholds to von Frey filaments were determined on hind paw prior to (Predose) and up to 8 weeks following i.p. injection of (50, 100 and 200 mg/Kg) Elder. **d** KMF group: paw withdrawal thresholds to von Frey filaments were determined on hind paw prior to (Predose) and up to 8 weeks following I.P. injection of (16, 32 and 64 mg/Kg) KMF. (NORM)normal non-diabetic untreated mice. *P ≤0.05 and **P ≤0.01 compared to vehicle (VEH) (*n* = 7 animals/group)

**Fig. 4:**
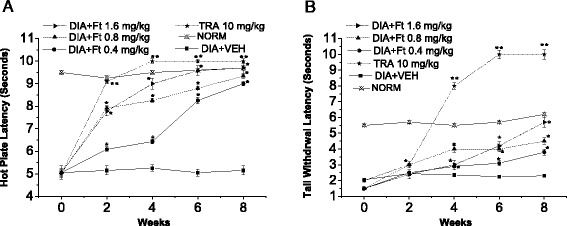
Effect of Ferutinin (Ft) and Tramadol (TRA) 10 mg/kg, as positive control, on the hot plate and tail withdrawal latencies in alloxan-induced diabetic mice. **a** Hot plate latency: (*Crossed-triangles, straight line*) NORM: normal control mice. (*Closed-squares, straight-line*) DIA + VEH: diabetic animals treated with vehicle as control. (*Solid-stars, dotted-line*) Positive control TRA 10 mg/kg: alloxan treated mice with TRA 10 mg/kg. (*Solid-circles, straight-line*) DIA + Ft 0.4 mg/kg: diabetic animals treated with Ft 0.4 mg/kg. (*Up-triangles, dashed-line*) DIA + Ft 0.8 mg/kg: diabetic animals treated with Ft 0.8 mg/kg. (*Right-triangles, dashed-dotted-line*) DIA + Ft 1.6 mg/kg: diabetic animals treated with Ft 1.6 mg/kg. **b** Tail withdrawal latency: (*Crossed-triangles, straight line*) NORM: normal control mice. (*Closed-squares, straight-line*) DIA + VEH: diabetic animals treated with vehicle as control. (*Solid-stars, dotted-line*) Positive control TRA 10 mg/kg: alloxan treated mice with TRA 10 mg/kg. (*Solid-circles, straight-line*) DIA + Ft 0.4 mg/kg: diabetic animals treated with Ft 0.4 mg/kg. (*Up-triangles, dashed-line*) DIA + Ft 0.8 mg/kg: diabetic animals treated with Ft 0.8 mg/kg. (*Right-triangles, dashed-dotted-line*) DIA + Ft 1.6 mg/kg: diabetic animals treated with Ft 1.6 mg/kg. Data are expressed in mean ± S.E.M. “*” means *P* < 0 .05 compared with vehicle. “**” means *P* < 0 .01 compared with vehicle

**Fig. 5:**
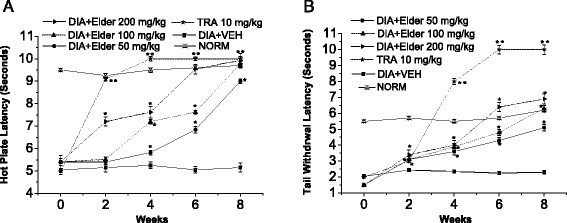
Effect of *S.nigra* aqueous extract (Elder) and Tramadol (TRA) 10 mg/kg, as positive control, on the hot plate and tail withdrawal latencies in alloxan-induced diabetic mice. **a** Hot plate latency: (*Crossed-triangles, straight line*) NORM: normal control mice. (*Closed-squares, straight-line*) DIA + VEH: diabetic animals treated with vehicle as control. (*Solid-stars, dotted-line*) Positive control TRA 10 mg/kg: alloxan treated mice with TRA 10 mg/kg. (*Solid-circles, straight-line*) DIA + Elder 50 mg/kg: diabetic animals treated with Elder 50 mg/kg. (*Up-triangles, dashed-line*) DIA + Elder 100 mg/kg: diabetic animals treated with Elder 100 mg/kg. (*Right-triangles, dashed-dotted-line*) DIA + Elder 200 mg/kg: diabetic animals treated with Elder 200 mg/kg. **b** Tail withdrawal latency: (*Crossed-triangles, straight line*) NORM: normal control mice. (*Closed-squares, straight-line*) DIA + VEH: diabetic animals treated with vehicle as control. (*Solid-stars, dotted-line*) Positive control TRA 10 mg/kg: alloxan treated mice with TRA 10 mg/kg. (*Solid-circles, straight-line*) DIA + Elder 50 mg/kg: diabetic animals treated with Elder 50 mg/kg. (*Up-triangles, dashed-line*) DIA + Elder 100 mg/kg: diabetic animals treated with Elder 100 mg/kg. (*Right-triangles, dashed-dotted-line*) DIA + Elder 200 mg/kg: diabetic animals treated with Elder 200 mg/kg. Data are expressed in mean ± S.E.M. “*” means *P* < 0 .05 compared with vehicle. “**” means *P* < 0 .01 compared with vehicle

**Fig. 6:**
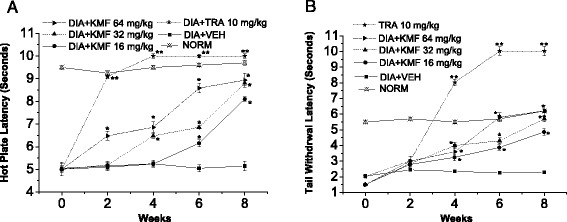
Effect of Kaempferol (KMF) and Tramadol (TRA) 10 mg/kg, as positive control, on the hot plate and tail withdrawal latencies in alloxan-induced diabetic mice. **a** Hot plate latency: (*Crossed-triangles, straight line*) NORM: normal control mice. (*Closed-squares, straight-line*) DIA + VEH: diabetic animals treated with vehicle as control. (*Solid-stars, dotted-line*) Positive control TRA 10 mg/kg: alloxan treated mice with TRA 10 mg/kg. (*Solid-circles, straight-line*) DIA + KMF 16 mg/kg: diabetic animals treated with KMF 16 mg/kg. (*Up-triangles, dashed-line*) DIA+ KMF 32 mg/kg: diabetic animals treated with KMF 32 mg/kg. (*Right-triangles, dashed-dotted-line*) DIA + KMF 64 mg/kg: diabetic animals treated with KMF 64 mg/kg. **b** Tail withdrawal latency: (*Crossed-triangles, straight line*) NORM: normal control mice. (*Closed-squares, straight-line*) DIA + VEH: diabetic animals treated with vehicle as control. (*Solid-stars, dotted-line*) Positive control TRA 10 mg/kg: alloxan treated mice with TRA 10 mg/kg. (*Solid-circles, straight-line*) DIA + KMF 16 mg/kg: diabetic animals treated with KMF 16 mg/kg. (*Up-triangles, dashed-line*) DIA+ KMF 32 mg/kg: diabetic animals treated with KMF 32 mg/kg. (*Right-triangles, dashed-dotted-line*) DIA + KMF 64 mg/kg: diabetic animals treated with KMF 64 mg/kg. Data are expressed in mean ± S.E.M. “*” means *P* < 0 .05 compared with vehicle. “**” means *P* < 0 .01 compared with vehicle

### Acute antidiabetic effect of Ferula and Elder extracts and their potential active isolates in alloxan-induced diabetic mice

The acute antidiabetic effect of various doses of Ferula extract and its active isolate, Ft, in diabetic animals is summarized in Table 1. All tested compounds results were statistically significant *p* < 0.05. The Ferula extract at all doses (12.5, 25 and 50 mg/kg) showed a significant effect compared to the control, with blood glucose levels dropping after 6 h of administration by 27.7, 43.4 and 43.1 %, respectively. GB, the positive control, prevented the drastic elevation of blood glucose after 1 h of glucose loading. Nevertheless, GB reduced the glucose level, 2 and 6 h after the glucose loading. Ft at all doses (0.4, 0.8 and 1.6 mg/kg) showed significant decrease in blood glucose level after 6 h dropping by 36.1, 34.1 and 51.2 %, respectively. Similarly, the acute antidiabetic effect of various doses of Elder extract and its most active isolate, KMF, in diabetic animals is summarized in Table 2. Elder extract showed significant decrease in blood glucose level after 6 h at all doses (50, 100 and 200 mg/kg) with the glucose level dropping by 41.1, 43.8 and 46.1 %, respectively. In case of KMF, at the lowest dose (16 mg/kg) showed a slight significant effect from that of control after 6 h of glucose administration. At higher doses (32 and 64 mg/kg) KMF showed a comparatively higher significant effects, with blood glucose levels dropping by 29.8 and 42.1 %, respectively compared to that of control after 6 h of glucose administration.

**Table 1:** Acute effect of *F.hermonis* EtAc extract (*Ferula*) and Ferutinin (Ft) on blood glucose of alloxan-induced diabetic mice (*n* = 7)

Group	Dose (mg/kg)	Mean blood glucose concentration ± S.E.M. (mg/dL)			
		0 h	0.5 h	2 h	6 h
Diabetic control	__	202.79 ± 5.60	211.93 ± 4.50	214.21 ± 9.70	210.15 ± 7.30
GB	5	219.70 ± 3.70	223.64 ± 1.80	159.74 ± 2.10	130.64 ± 2.40**
*Ferula*	12.5	210.65 ± 3.30	229.50 ± 3.10	118.77 ± 3.40	152.33 ± 3.60*
*Ferula*	25	213.44 ± 2.90	244.78 ± 3.10	146.33 ± 2.60	120.77 ± 2.40*
*Ferula*	50	206.33 ± 3.10	147.56 ± 2.80	136.99 ± 2.90	117.42 ± 1.80*
Ft	0.4	210.41 ± 3.10	176.25 ± 2.40	141.66 ± 2.20	134.50 ± 3.50*
Ft	0.8	211.33 ± 3.20	174.55 ± 2.80	153.55 ± 2.40	139.33 ± 2.60*
Ft	1.6	217.90 ± 3.50	126.56 ± 2.10	116.89 ± 2.60	106.40 ± 2.20*

*S.E.M.* standard error of the mean

*p < 0.05 significant from the control animals

**p < 0.01 significant from the control animals

**Table 2:** Acute effect of *S.nigra* aqueous extract (Elder), Kaempferol (KMF) on blood glucose of alloxan-induced diabetic mice (*n* = 7)

Group	Dose (mg/kg)	Mean blood glucose concentration ± S.E.M. (mg/dL)			
		0 h	0.5 h	2 h	6 h
Diabetic control	__	202.79 ± 5.60	211.93 ± 4.50	214.21 ± 9.70	210.15 ± 7.30
GB	5	219.70 ± 3.70	223.64 ± 1.80	159.74 ± 2.10	130.64 ± 2.40**
Elder	50	202.54 ± 3.30	189.91 ± 3.10	98.81 ± 1.40*	119.35 ± 1.90
Elder	100	203.90 ± 2.60	175.83 ± 1.90	124.81 ± 1.80	114.57 ± 1.70*
Elder	200	204.54 ± 2.10	138.91 ± 2.80	128.90 ± 1.40	110.35 ± 1.90*
KMF	16	217.34 ± 1.80	208.82 ± 2.10	200.11 ± 3.10	182.54 ± 1.50*
KMF	32	214.13 ± 1.70	201.23 ± 2.70	174.70 ± 1.10	150.43 ± 2.40*
KMF	64	211.68 ± 1.60	200.57 ± 1.60	169.55 ± 2.70	122.65 ± 2.10*

*S.E.M.* standard error of the mean

*p < 0.05 significant from the control animals

**p < 0.01 significant from the control animals

### Subchronic effect of the Ferula and Elder extracts and their potential active isolates in alloxan-induced diabetic mice

The blood glucose levels of diabetic control mice were significantly higher than those of the control (negative control) mice during the experiment period as shown in Tables 3 and 4. All tested compounds results were statistically significant *p* < 0.05. The highest reduction in blood glucose using Ferula extract was observed with a dose of 50 mg/kg, showing 47.1 % reduction in blood glucose levels on the 8th day compared with 37.1 and 39.4 % reduction in case of 25, 50 mg/kg doses respectively.

**Table 3:** Subchronic effect of *F.hermonis* EtAc extract (*Ferula*), Ferutinin (Ft) on blood glucose of alloxan-induced diabetic mice (*n* = 7)

Group	Dose (mg/kg)	Mean blood glucose concentration ± S.E.M. (mg/dL)			
		1st day	3rd day	5th day	8th day
Control	__	107.90 ± 2.50	109.80 ± 3.60	108.56 ± 3.20	109.50 ± 3.70
Diabetic control^a^	__	207.79 ± 5.60***	214.93 ± 4.50***	209.91 ± 9.70***	210.55 ± 7.30***
GB^b^	5	186.70 ± 3.70	179.53 ± 2.90	161.54 ± 2.40**	171.97 ± 3.10
*Ferula*	12.5	162.67 ± 2.60	152.33 ± 3.10	142.69 ± 2.02	132.53 ± 3.60*
*Ferula*	25	143.34 ± 1.90	134.88 ± 3.10	134.48 ± 2.60	127.55 ± 2.40*
*Ferula*	50	142.12 ± 1.30	138.56 ± 2.80	112.66 ± 1.90	111.42 ± 1.60*
Ft	0.4	144.11 ± 1.20	134.89 ± 3.10	155.32 ± 2.50	136.13 ± 1.30*
Ft	0.8	120.33 ± 1.70	148.52 ± 2.70	140.44 ± 2.70	132.33 ± 1.50*
Ft	1.6	125.21 ± 2.90	142.56 ± 2.10	137.89 ± 2.60	122.13 ± 1.60*

*S.E.M.* standard error of the mean

*p < 0.05 significant from the control animals

**p < 0.01 significant from the control animals

***p < 0.001 significant from the control animals

^a^Compared to vehicle control

^b^Compared to diabetic control

**Table 4:** Subchronic effect of *S.nigra* aqueous extract (Elder), Kaempferol (KMF) on blood glucose of alloxan-induced diabetic mice (*n* = 7)

Group	Dose (mg/kg)	Mean blood glucose concentration ± S.E.M. (mg/dL)			
		1st day	3rd day	5th day	8th day
Control	__	107.90 ± 2.50	109.80 ± 3.60	108.56 ± 3.20	109.50 ± 3.70
Diabetic control^a^	__	207.79 ± 5.60***	214.93 ± 4.50***	209.91 ± 9.70***	210.55 ± 7.30***
GB^b^	5	186.70 ± 3.70	179.53 ± 2.90	161.54 ± 2.40**	171.97 ± 3.10
Elder	50	143.18 ± 1.30	127.89 ± 3.10	119.32 ± 2.50*	123.13 ± 1.30
Elder	100	144.17 ± 1.70	128.52 ± 2.70	124.44 ± 2.70	113.33 ± 1.50*
Elder	200	142.12 ± 1.50	122.76 ± 2.10	120.89 ± 2.60	110.13 ± 1.60*
KMF	16	156.86 ± 2.10	153.86 ± 2.30	151.34 ± 1.10	149.67 ± 2.90*
KMF	32	140.30 ± 2.40	141.30 ± 2.80	142.60 ± 1.30	139.41 ± 2.20*
KMF	64	142.68 ± 1.40	137.25 ± 1.70	132.55 ± 2.00	128.65 ± 2.50*

*S.E.M.* standard error of the mean

*p < 0.05 significant from the control animals

**p < 0.01 significant from the control animals

***p < 0.001 significant from the control animals

^a^Compared to vehicle control

^b^Compared to diabetic control

The Ft at all doses (0.4, 0.8 and 1.6 mg/kg) showed significant decrease in blood glucose level on the 8th day compared to that of the diabetic control with the mice glucose levels dropping by 35.3, 37.2 and 42.0 %, respectively. Similarly, Elder extract at all doses (50, 100 and 200 mg/kg) showed significant decrease in blood glucose level on the 8th day dropping by 41.5, 46.2 and 47.7 %, respectively. KMF showed a significant effect compared to that of diabetic control on the 8th day at all doses (16, 32 and 64 mg/kg), with blood glucose levels dropping by 28.9, 33.8 and 38.9 %, respectively.

During the subchronic administration, mice treated with Ferula and Elder extracts and their potential active isolates, were also monitored for changes in weight (Tables 5 and 6). The Ferula extract showed 2.7, 2.2 and 13.5 % increase in body weight at doses of 12.5, 25 and 50 mg/kg respectively on the 8th day. In case of Ft at doses (0.4, 0.8 and 1.6 mg/kg), showed 7.1, 5.0 and 11.6 % increase in body weight on the 8th day, respectively. Similarly, Elder extract at doses (50, 100 and 200 mg/kg) showed 10.3, 9.5 and 13.0 % increase in body weight on the 8th day. Also, KMF at all doses (16, 32 and 64 mg/kg) showed a significant increase in body weight on the 8th day, of 10.4, 12.5 and 16.0 % respectively.

**Table 5:** *Subchronic effect* of *F.hermonis* EtAc extract (*Ferula*), Ferutinin (Ft) on body weights in alloxan-induced diabetic mice (*n* = 7)

Group	Dose (mg/kg)	Mean body weight ± S.E.M. (gm)			
		1st day	3rd day	5th day	8th day
Control	__	25.90 ± 0.50	26.00 ± 0.60	26.01 ± 0.97	25.09 ± 0.70
Diabetic control^a^	__	28.68 ± 0.70	27.10 ± 0.20	27.15 ± 0.80	27.10 ± 0.50
GB^b^	5	22.90 ± 0.70	28.17 ± 1.70	28.54 ± 0.40	30.37 ± 1.10*
*Ferula* ^b^	12.5	26.00 ± 1.30	26.60 ± 1.70	26.70 ± 2.00	26.70 ± 1.20*
*Ferula* ^b^	25	27.20 ± 1.70	27.50 ± 1.80	27.50 ± 1.80	27.80 ± 2.20*
*Ferula* ^b^	50	25.10 ± 1.50	27.20 ± 2.10	27.54 ± 1.90	28.50 ± 2.40*
Ft^b^	0.4	25.31 ± 1.80	26.80 ± 1.50	26.90 ± 1.60	27.10 ± 1.90*
Ft^b^	0.8	27.00 ± 1.70	28.10 ± 2.10	28.30 ± 1.30	28.33 ± 2.10*
Ft^b^	1.6	28.50 ± 1.90	29.60 ± 2.50	30.10 ± 2.40	31.80 ± 2.80*

*S.E.M.* standard error of the mean

*p < 0.05 significant from the control animals

^a^Compared to vehicle control

^b^Compared to diabetic control

**Table 6:** *Subchronic effect* of *S.nigra* aqueous extract (Elder), Kaempferol (KMF) on body weights in alloxan-induced diabetic mice (*n* = 7)

Group	Dose (mg/kg)	Mean body weight ± S.E.M. (gm)			
		1st day	3rd day	5th day	8th day
Control	__	25.90 ± 0.50	26.00 ± 0.60	26.01 ± 0.97	25.09 ± 0.70
Diabetic control^a^	__	28.68 ± 0.70	27.10 ± 0.20	27.15 ± 0.80	27.10 ± 0.50
GB^b^	5	22.90 ± 0.70	28.17 ± 1.70	28.54 ± 0.40	30.37 ± 1.10*
Elder^b^	50	27.10 ± 1.30	28.90 ± 3.10	29.52 ± 2.10	29.91 ± 1.20*
Elder^b^	100	27.50 ± 1.70	27.83 ± 2.70	28.90 ± 1.70	30.10 ± 1.50*
Elder^b^	200	27.00 ± 1.50	27.50 ± 2.10	28.10 ± 1.50	30.50 ± 1.40*
KMF^b^	16	20.50 ± 1.50	21.80 ± 1.30	22.45 ± 1.10	22.64 ± 1.70*
KMF^b^	32	22.33 ± 1.30	22.81 ± 1.70	23.91 ± 1.40	25.13 ± 1.20*
KMF^b^	64	22.11 ± 1.60	22.94 ± 1.80	23.54 ± 1.80	25.64 ± 1.20*

*S.E.M.* standard error of the mean

*p < 0.05 significant from the control animals

^a^Compared to vehicle control

^b^Compared to diabetic control

In order to evaluate *in-vivo* antioxidant effect of the tested extracts and compounds, CAT level in serum of each mouse was monitored on 1st, 3rd, 5th and 8th days after administration (Tables 7 and 8). As shown in Table 7, diabetic mice were monitored for changes in serum CAT level after treatment with GB and various doses of the Ferula and Ft. The Ferula extract at doses of 12.5, 25 and 50 mg/kg had a gradual rise in serum CAT activity to reach a significant difference on 8th day (2.1, 3.2 and 9.5 % respectively) as compared with diabetic control mice. Ft at all doses (0.4, 0.8 and 1.6 mg/kg) had a gradual rise in serum CAT activity to reach significant differences on 8th day (10.0, 13.1 and 13.9 %, respectively) as compared with diabetic control mice. Similarly, as shown in Table 8, Elder extract had a gradual rise in serum CAT activity to reach a significant difference on the 8th day with CAT activities of 3.0, 4.0 and 9.5 % for doses (25, 50 and 100 mg/kg), respectively. KMF at all doses (16, 32 and 64 mg/kg) had also shown a gradual rise in serum CAT activity to reach a significant difference on the 8th day (18.6, 26.4 and 29.4 %, respectively) as compared with diabetic control mice.

**Table 7:** *In vivo* assessment of the antioxidant activity of *F.hermonis* EtAc extract (*Ferula*), Ferutinin (Ft) using catalase levels in serum of alloxan-induced diabetic mice (*n* = 7)

Group	Dose (mg/kg)	Catalase level ± S.E.M. (kU/I)			
		1st day	3rd day	5th day	8th day
Control	__	41.00 ± 1.50	41.50 ± 1.60	40.86 ± 1.20	41.62 ± 1.70
Diabetic control^a^	__	23.67 ± 1.60***	24.93 ± 1.30***	23.01 ± 1.90***	22.02 ± 1.40***
GB^b^	5	22.60 ± 1.70	25.00 ± 1.70	31.54 ± 1.40*	32.37 ± 1.00**
*Ferula* ^b^	12.5	23.31 ± 1.40	22.48 ± 3.10	23.00 ± 2.50*	23.80 ± 1.60**
*Ferula* ^b^	25	23.17 ± 1.70	21.05 ± 2.10	23.20 ± 2.30*	23.90 ± 1.30**
*Ferula* ^b^	50	23.02 ± 1.60	23.17 ± 2.10	24.30 ± 2.60*	25.20 ± 1.30*
Ft^b^	0.4	20.10 ± 1.20	19.70 ± 1.10	21.00 ± 1.60*	22.10 ± 1.20*
Ft^b^	0.8	21.05 ± 1.50	21.58 ± 1.60	23.35 ± 1.80*	23.80 ± 0.80*
Ft^b^	1.6	22.50 ± 1.80	22.80 ± 1.40	23.91 ± 1.10*	25.62 ± 1.20*

*S.E.M.* standard error of the mean

*p < 0.05 significant from the control animals

**p < 0.01 significant from the control animals

***p < 0.001 significant from the control animals

^a^Compared to vehicle control

^b^Compared to diabetic control

**Table 8:** *In vivo* assessment of the antioxidant activity of *S.nigra* aqueous extract (Elder), Kaempferol (KMF) using catalase levels in serum of alloxan-induced diabetic mice (*n* = 7)

Group	Dose (mg/kg)	Catalase level ± S.E.M. (kU/I)			
		1st day	3rd day	5th day	8th day
Control	__	41.00 ± 1.50	41.50 ± 1.60	40.86 ± 1.20	41.62 ± 1.70
Diabetic control^a^	__	23.67 ± 1.60***	24.93 ± 1.30***	23.01 ± 1.90***	22.02 ± 1.40***
GB^b^	5	22.60 ± 1.70	25.00 ± 1.70	31.54 ± 1.40*	32.37 ± 1.00**
Elder^b^	50	26.81 ± 1.40	25.98 ± 3.10	26.50 ± 2.50	27.60 ± 1.60*
Elder^b^	100	25.77 ± 1.70	24.25 ± 2.10	25.70 ± 2.30	26.80 ± 1.20*
Elder^b^	200	26.22 ± 1.60	26.67 ± 2.30	27.80 ± 2.60	28.70 ± 1.30*
KMF^b^	16	24.22 ± 0.60	23.94 ± 0.30	26.00 ± 1.00	28.74 ± 1.10*
KMF^b^	32	22.33 ± 1.10	23.41 ± 1.20	25.91 ± 1.40	28.23 ± 1.20*
KMF^b^	64	24.20 ± 0.70	25.55 ± 0.90	30.05 ± 0.70	31.32 ± 1.30*

*S.E.M.* standard error of the mean

*p < 0.05 significant from the control animals

**p < 0.01 significant from the control animals

***p < 0.001 significant from the control animals

^a^Compared to vehicle control

^b^Compared to diabetic control

### Management of diabetic neuropathy

Declining of peripheral nerve conduction is an important indicator for diabetic patients having peripheral neuropathy [36–38]. Therefore, we examined the effect of Ferula extract, Ft, Elder extract and KMF treatment on sensory function by measuring the thermal latency with tail flick and hot plate tests and tactile allodynia using von Frey filaments on the 8th week after alloxan injection.

Treatment of the alloxan-induced diabetic mice with Ferula extract markedly improved the thermal latency compared with TRA 10 mg/kg positive control (Fig. 2a). Diabetic mice exhibited temporary hyperalgesic response in thermal tests. On the 8th week after alloxan injection, treatment with Ferula extract showed a marked improvement in hot-plate latency compared to vehicle treated group by 49.5, 62.1 and 69.6 % in doses of 12.5, 25 and 50 mg/kg, respectively (Fig. 2a).

Nevertheless, treatment with all doses of Ferula extract on the 8th week after alloxan injection, demonstrated a marked improvement in tail-flick latency by ca. 0.4, 0.69 and 0.73 folds for doses of 12.5, 25 and 50 mg/kg, respectively, compared to vehicle treated group (Fig. 2b).

Furthermore, on the 8th week, treatment with all doses (12.5, 25 and 50 mg/kg) of Ferula extract markedly improved tactile allodynia utilizing von Frey filaments by 1.8, 3.1 and 5.1 folds respectively, compared to vehicle treated group (Fig. 3a).

Additionally, treatment with all doses (0.4, 0.8 and 1.6 mg/kg), on the 8th week, of Ft markedly improved hot-plate latency by 74.8, 80.6 and 88.3 %, respectively, compared to vehicle treated group (Fig. 4a). Nonetheless, treatment with all doses of Ft, the tail-flick latency have markedly improved by ca. 0.5, 1.0 and 1.5 folds in Ft doses (0.4, 0.8 and 1.6 mg/kg), respectively (Fig. 4b).

On the 8th week, treatment with all doses (0.4, 0.8 and 1.6 mg/kg) of Ft extract significantly improved tactile allodynia utilizing von Frey filaments by 1.8, 2.7 and 4.9 folds respectively, compared to vehicle treated group (Fig. 3b).

Moreover, treatment with Elder extract in all doses (50, 100 and 200 mg/kg) markedly improved hot-plate latency by 74.8, 89.9 and 92.8 %, respectively, and tail-flick latency by ca. 1.2, 1.7 and 2.0 folds (Fig. 5a and b), and von Frey filaments by 2.9, 4.9 and 7.8 folds respectively, compared to vehicle treated groups (Fig. 3c).

In addition, treatment with KMF (16, 32 and 64 mg/kg) showed a marked improvement in hot-plate latency by 57.3, 70.9 and 72.8 %, and tail-flick latency by ca. 1.1, 1.5 and 1.7 folds, respectively, compared to vehicle treated group (Fig. 6a-b), and von Frey filaments by 2.4, 4.1 and 5.9 folds respectively, compared to vehicle treated groups (Fig. 3d).

## Discussion

The aim of the present work involves the study of possible *in vivo* antioxidant, and antihyperglycemic and related complications management by Ferula, Elder and their potential active isolates. Extended exposure to hyperglycemia induces the development of microvascular and macrovascular complications associated with DM [39]. The high oxidative stress in diabetics significantly contributes to the complications of this disease [40] and free radicals excessive production is a discovered phenomenon associated with diabetic complications [41]. Amelioration of DM and diabetic complications with minimal side effect is still a major challenge to the health system [39].

Consequently, wider investigation of potent natural antidiabetics with fewer side effects was the aim of many scientists worldwide. In this study we chose Ferula and Elder extracts based on its folkloric use in treatment of many chronic diseases in Lebanon [9, 42].

In the present work, bio-guided fractionation utilizing column chromatography, TLC and RP-HPLC, indicated that Ft and KMF is the most effective compounds in Ferula and Elder extracts, respectively. However, the extracts apparently showed a more potent pattern of amelioration of DM and DN than the isolated compounds, especially in longer duration of treatment. This may be attributed to synergistic pattern of augmentation between the most active compounds and other compounds in the extracts [25].

The extracts and isolated compounds had a dose dependent effect in hyperglycemic mice, with significant decrease of blood glucose levels at the highest dose levels.

It has been found that the highest dose levels of Ferula extract (50 mg/kg), Ft (1.6 mg/kg), Elder (200 mg/kg) along with KMF (64 mg/kg) are the most effective doses in the acute and subchronic groups. Compared to that of the synthetic drug, GB, these doses have more significant effect on blood glucose level. During testing the acute antidiabetic effect, an initial increase in blood glucose levels was observed during the first 0.5 h after IP administration of Ferula and Elder extracts. This initial temporary hyperglycemia may be due to pre-glucose loading, whilst, the extracts did not start to give their effect directly after administration, as reported before in literature [26].

In the acute antidiabetic effect, Ft had shown a relatively higher reduction in glucose level compared with other tested compounds. This could be advantageous in fast reduction of blood sugar level.

In the subchronic antidiabetic effect, Elder extract had shown a relatively higher reduction in glucose level compared to other tested compounds. An effect that could be beneficial for designing a long acting therapy for controlling DM.

Ferula extract, Ft, Elder extract and KMF showed a significant elevation in body weight, as an evidence of amelioration of hyperglycemia, as demonstrated before with medications used in management of DM [43, 44].

Recently, much attention has been given on the role of oxidative stress as the key and common event in the pathogenesis of different diabetic complications [45].

In the current study, the activity of CAT reduced in diabetic mice as reported earlier [45, 46]. This could be the result of inactivation caused by alloxan-generated reactive oxygen species (ROS). Subchronic treatment of DM with all doses, especially with the highest dose of Ferula extract, Ft, Elder extract and KMF could have reversed the activities of this enzymatic antioxidant, which might be attributed to lessened oxidative stress as evidenced by the elevation in CAT activity.

In acute case, the effect of Ft in the same concentration relative to that present in the Ferula extract, is comparatively showing equi-potent antihyperglycemic and subchronic antioxidant activity to Ferula extract, which may indicate that Ft is the most effective bioactive component in Ferula extract.

Likewise, the effect of KMF in the same concentration relative to that present in the Elder extract, is comparatively showing equi-potent acute antihyperglycemic and antioxidant activity to Elder extract, which may indicate that KMF is the most effective bioactive compound in Elder extract.

The present study describes for the first time the anti-hyperalgesic activity of Ferula and Elder extracts in an alloxan-induced diabetic mouse model. Administration of Ferula extract, Ft, Elder extract and KMF also alleviated hyperalgesia in pain conditions compared to that of the positive control, tramadol (TRA).

The data shows that Ferula and Elder extracts are highly effective against thermal hyperalgesia and tactile allodynia in animal models of neuropathic pain. On the other hand, these extracts differ in their profile of activity with respect to their active compounds and modality.

Ft has shown a similar profile of activity like Ferula extract, with more potent anti-hyperalgesic activity in the alloxan-induced diabetic mice compared to the parent extract.

Furthermore, KMF had demonstrated a similar pattern of activity like Elder extract, with less potent antinociceptive activity in the alloxan-induced diabetic mice compared to the Elder extract. The differences in potency was observed before with other natural, like rutin, and synthetic compounds, like carbamazepine [25, 33].

Elder extract was the most active against tactile allodynia producing ca. 6 folds improvement following a single administration in the alloxan-induced diabetic mice, but required repeated administration to achieve its maximal effect against mechanical hyperalgesia, with a relatively similar efficacy to that observed with TRA. Moreover, hypoglycemia was not reported with any of the test compounds, and the incidence of other adverse events was not significant between the groups.

These findings provide health professionals with a promising natural remedies intended for symptomatic amelioration of diabetic neuropathy, a safe [14, 47] antidiabetic agent as well as micro- and macrovascular complications management of DM with lower side effects.

Relatively rapid onset medication (Ft) may be better for acute management, while slower ones (Elder extract) may be more beneficial in long term management. Additionally, clinical studies should follow this study to give reliable information for choosing between alternative therapies.

## Conclusion

In conclusion, the present study indicated that Ferula and Elder extracts exerted remarkable hypoglycemic activity and improved peripheral nerve function, which might be due to Ft and KMF, respectively, that prevents oxidative stress in diabetic animals. Therefore, the observed *in-vivo* antioxidant potential of Ferula and Elder extracts might possibly be one of the mechanisms of action responsible for their antinociceptive effect.

Taken together with the known improved safety profile of the tested compounds, these data indicate their benefit in the treatment of neuropathic pain conditions without the use-limiting side-effects inherent with existing therapies.

## Abbreviations

DM: Diabetes mellitus
EtAc: Ethyl acetate
Ferula: *Ferula hermonis* EtAc extract
i.p.: Intraperitoneal
Elder: *Sambucus nigra* aqueous extract
RP-HPLC: Reversed phase-high performance liquid chromatography
DMSO: Dimethyl sulfoxide
LD50: Lethality towards 50 % of a population
ca.: Approximately
GB: Glibenclamide
TRA: Tramadol
ROS: Reactive oxygen species
CAT: Serum catalase level
